# DUSP1 promotes muscle atrophy by inhibiting myocyte differentiation in cachectic patients

**DOI:** 10.3389/fonc.2022.1040112

**Published:** 2022-11-01

**Authors:** Xiangyu Sui, Xiangyu Mao, Guohao Wu, Qingyang Meng

**Affiliations:** Department of General Surgery, Zhongshan Hospital of Fudan University, Shanghai, China

**Keywords:** cancer cachexia, skeletal muscle, muscle atrophy, myogenesis, DUSP1

## Abstract

**Background:**

Skeletal muscle atrophy is the major hallmark of cancer cachexia. The mechanisms underlying muscle wasting remain elusive in cachectic patients. Our research seeks to identify differentially expressed genes (DEGs) between non-cachectic and cachectic cancer patients and elucidate their functions.

**Methods:**

We screened the DEGs of skeletal muscle between patients with and without cachexia from microarray data. Biological function of DEGs is analyzed through gene enrichment analysis, while an interaction network is constructed to visualize how genes are related. A Spearman’s correlation analysis demonstrated the clinical significance of DUSP1 related to cancer cachexia. Skeletal muscle samples were collected and histomorphology studies were conducted. Function of DUSP1 on myogenesis was clarified by qPCR, western blotting, and immunofluorescence.

**Results:**

We screened 324 DEGs in skeletal muscle from patients with and without cachexia. The results of the gene enrichment analysis indicated that inflammatory cytokines and immune responses contribute significantly to the pathological condition of cachexia. DUSP1 was one of the key genes in the regulating network. DUSP1 protein and mRNA levels were increased significantly in skeletal muscle tissues from patients with cancer cachexia. DUSP1 expression in cachectic group was found to have negative correlation with SMA, prealbumin and BMI and positive correlation with TNFα, IL6 and weight loss. Significant changes of myogenesis related genes were observed in myocyte after DUSP1 was overexpressed and knocked down.

**Conclusion:**

In skeletal muscle of cachectic patients, DUSP1 expression was observed to be higher and thus DUSP1 promote muscle atrophy by inhibiting myogenesis. DUSP1 is expected to be a specific target in cancer cachexia for preventing and treating muscle atrophy.

## Introduction

Cachexia is a metabolic syndrome with multifunctionality characterized by involuntary weight loss as a result of skeletal muscle wasting and/or degradation of adipose tissue, which approximately 30%–90% of cancer patients suffered from, preferentially at advanced tumor stages (1). Progressive impairment and dysfunction of multiple organs are caused by cachexia that is responsible for approximately 20% of all cancer mortalities (2). Cancer cachexia remains unclear in terms of its pathophysiology, but the consensus is that there is a complex interaction between tumor and patient-derived factors which result in a negative energy balance (3). Accordingly, identifying crucial molecules that contribute to the pathogenic process of cancer cachexia could facilitate the formulation of therapeutic strategies for preventing or alleviating cachexia.

Approximately 40-50% of a person’s body weight is made up of skeletal muscle, which is both the body’s largest tissue mass and its primary protein storage organ (4). An important characteristic of cachexia is the decrease in body weight and skeletal muscle wasting, whether there is a decrease in adipose tissue associated with them or not, altered metabolism and chronic inflammation (5). It is suggested that cachexia appears to be the leading cause of death among cancer patients due to muscle wasting (6). Thus, maintaining muscle mass may increase survival rates. It has been well established at the cellular level that muscle atrophy in cachexia is predominantly caused by myofiber atrophy, the size of which is controlled by the ratio of protein production to breakdown (7). Previous research revealed that expression of genes according to a common program underlies multiple forms of skeletal muscle atrophy (8). It was demonstrated as a standard response that transcriptional activation of ubiquitin ligases occurs in skeletal muscle atrophy resulting from cancer cachexia (9). Hence, it is essential to better understand the pathophysiological basis of skeletal muscle atrophy, which represents a major clinical feature of cachexia.

Dual specificity phosphatase (DUSP) can dephosphorylate and inactivate MAPKs, ERK, p38, and JNK in multiple manners as mitogen-activated protein kinase (MAPK) phosphatases (10). As a member of the MKP family, mitogen-activated protein kinase phosphatase-1 (MKP-1) is another name for DUSP1 (11). DUSP1 participates in numerous biological processes, for instance, inflammation, cellular physiological activity, neuroprotection and neuronal axonal development and by regulating MAPK signaling pathways (12). DUSP1 overexpression in prostate cancer cells promotes apoptosis through inhibiting the MAPK/NF-kB signaling pathway (13). In osteoarthritis, miR-337-3p inhibits the ubiquitination of DUSP1 through negative targeting of SKP2 and inactivates the p38 MAPK pathway (14). In addition, DUSP1 has been proved to acts *via* MAPK signaling pathway to induce the mRNA destabilization of protein-tristetraprolin to inhibit secretion of pro-inflammatory cytokine from smooth muscle cells (15). However, its roles in skeletal muscle and whether it can regulate the cellular activities of muscle cells in pathological process of cachexia remains unknown.

We aimed to discover the expression level of DUSP1 in cachectic patients and its potential functions in our study by the following three steps: First of all, expression profile data of cachectic and non-cachectic samples in the Gene Expression Omnibus (GEO) was acquired to analyze the differentially expressed genes (DEGs). Subsequently, functional enrichment analysis was then conducted using the protein-protein interaction (PPI) network, GSEA, KEGG and GO. Furthermore, cellular functions and clinical significance of DUSP1 in cachexia were investigated.

## Materials and methods

### Human tissue specimens

This study enrolled 97 patients diagnosed with a primary gastrointestinal tumor who underwent radical resection at the General Surgery Department, Zhongshan Hospital, Fudan University. Cancer cachexia was diagnosed in 49 patients with a six-month weight loss of more than 5%; there was no significant weight loss among the rest of them during the past six months. For purpose of determining the skeletal muscle area (SMA, cm^2^), we acquired CT image of the plane of the third lumbar vertebrae for analysis. No radiotherapy or chemotherapy was administered prior to surgery. In all patients, total resections were performed, and histopathological and pathological analysis was performed between 2018.06 and 2020.06. Muscles were derived from the abdominal incision site on rectus abdominis. All tissues were instantly frozen and preserved in liquid nitrogen until use. Regulatory approval of the study was obtained from Zhongshan Hospital, Fudan University (Approval No. B2019-193R). Informed consent was obtained from all participants prior to conducting in the study.

### Cell culture and differentiation

Mouse derived C2C12 cells were kindly provided by Prof. Ying Feng from Shanghai Institute of Nutrition and Health, Chinese Academy of Sciences. C2C12 cells were cultured in Dulbecco’s Modification of Eagle’s Medium (DMEM) supplemented with 10% fetal bovine serum (FBS) and maintained in a humidified incubator with 5% CO2 at 37°C. To differentiate C2C12 myoblasts into myotubes, myoblasts were grown in DMEM supplemented with 10% FBS until they reached ~85–90% confluence. The FBS medium was then replaced with DMEM medium supplemented with 2% horse serum. Cells were incubated for 4 additional days to allow for terminal differentiation.

### Data acquisition

The transcriptome data and clinical information was obtained from Gene Expression Omnibus (GEO, ID: GSE130563). This dataset included expression data obtained from rectus abdominis biopsies of patients undergoing radical resection surgery with or without cachexia.

### Functional enrichment analysis

Differentially expressed genes (DEGs) analysis was conducted through the limma package of R software between cachectic and non-cachectic groups. The significance criteria were | logFC | > 0.4 and P-value < 0.05. Gene Ontology (GO) functional analysis and Kyoto Encyclopedia of Genes and Genomes (KEGG) pathway analysis were implemented using the clusterProfiler package of R software. Gene set enrichment analysis (GSEA) was implemented by GSEA software (version 4.1.0, www.gsea-msigdb.org/gsea).

### Protein-protein interaction network (PPI network)

PPI network of DEGs was constructed through Online program STRING (string-db.org/). Visualization of the interaction results from the tool was constructed through Cytoscape software (v3.7.2).

### Transcription factor (TF-gene) and gene-miRNA interaction networks

TF-gene and gene-miRNA networks were constructed based on data from ENCODE ChIP-seq (https://www.encodeproject.org/) and miRTarBase v8.0 (mirtarbase.cuhk.edu.cn/php/download.php), respectively.

### Quantitative real-time PCR

We extracted total RNA from muscles by TRIzol Reagent (ThermoFisher), following instructions provided by the manufacture. The cDNA was synthesized according to the reverse transcription kit. Previously, quantitative real-time PCR protocols were described (8). GAPDH was used as normalized controls. Further information regarding primers is provided in **Supplementary Table 1**. Analysis of gene expression correlations was calculated through the 2^-△△Ct^ method. Each experiment was repeated in triplicate.

### Construction of plasmid and small interfering RNAs (siRNAs)

The design and synthesis of the DUSP1 overexpression plasmid pEX-3/DUSP1 and empty plasmid pEX-3 were completed by GenePharma company (Shanghai, China). The design of siRNAs targeting DUSP1 were completed by GenePharma. The Lipofectamine 2000 and Lipofectamine RNAiMAX (Invitrogen, USA) were used in the cell transfection procedure described previously (8).

### Immunoblot

Total protein extracts were obtained and analyzed by western blotting in accordance with our previous description (8). DUSP1 (GeneTex, GTX47608, 1:1000), myogenic differentiation 1 (MyoD1) (Sigma, M6190, 1:1000), and myogenin (MyoG) (Abcam, ab124800, 1:500) were used as primary antibodies. The expression of tubulin was used as an endogenous control. Each experiment was repeated in triplicate.

### HE staining

Incubation of muscle tissue was conducted in hematoxylin solution (Sigma) for 15 min and rinsed to remove excess stain solution. Then the sections were immersed in eosin B solution (Sigma) for 2 minutes and rinsed twice quickly using distilled water. For dehydration, section was immersed in various concentrations of ethanol and xylane (70%, 95% and 100% ethanol for 30s; xylane for 60s). All microscopy quantifications were performed in three sections per sample. Cross sectional area (CSA) quantification was conducted by ImageJ (National Institutes of Health, USA).

### Immunohistochemistry

Isolated muscles were fixed in 4% paraformaldehyde (PFA). Fixation of tissue sections was performed in methanol at -20°C for 10 minutes. The tissue sections were rinsed in phosphate buffered saline (PBS) for three times, incubated in hydrogen peroxide solution (0.3% H2O2 in PBS) for 5 minutes for the removal of endogenous peroxidases, and rinsed again in PBS. After cooling for 1 hour, tissue sections were blocked at room temperature with 5% normal goat serum in blocking buffer for 45 minutes. After that, incubation of slides in dilutions of DUSP1 (1:200) in blocking reagent was performed overnight at 4°C. Then incubation of slides in a second primary antibody was performed for 1 h followed by rinsing in PBS/0.1% Tween-20. All microscopy quantifications were performed using three sections per sample. Percentage of DUSP1 positive fibers was measured using ImageJ.

### Immunofluorescence of cultured cells

Incubation of C2C12 cells was performed on etched glass coverslips following the methods in the literature (16). Cells were washed and fixed in cold methanol for 20 minutes after the coverslips were rinsed in PBS. After three PBS washings, the slides were washed in 5% BSA for 30 min for blocking. The Myosin heavy chain (MyHC) (Sigma, M4276) dilution was incubated overnight at 4°C. Following PBS rinse, all coverslips were incubated with secondary antibody and MitoTracker Green FM (YEASEN, Shanghai) fluorescence probe at room temperature for 1 hour. Rinsing coverslips with PBS again. Images were taken by DM2500 Fluorescence Microscope (Leica).

### Statistical analysis

Statistical analysis was completed using GraphPad Prism. Data characteristics were expressed as the mean ± standard deviation of independent samples. Differences between groups were determined using a student’s t-test. Spearman correlation coefficients were conducted to assess relationships between continuous variables. *t*-test and χ^2^ test were conducted respectively for continuous data and categorical data. P values of less than 0.05 were considered to be significant. * stands for *p*< 0.05 and ns stands for not significant.

## Results

### Identification and functional enrichment analysis of DEGs in muscle samples with or without cachexia

**Figure 1:**
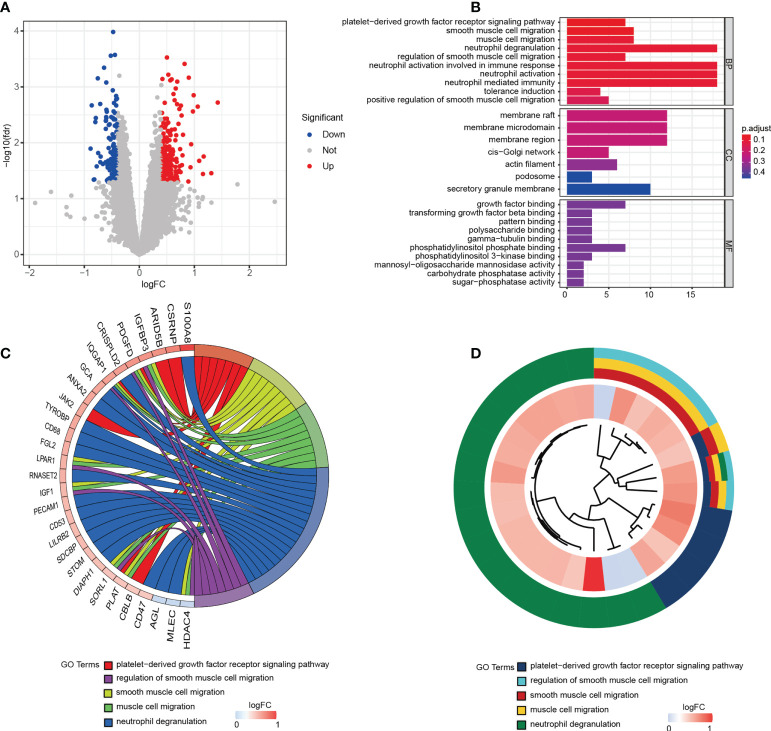
324 DEGs were identified as being statistically significant between groups. **(A)** Volcano plot of DEGs. **(B)** GO functional enrichment analysis of DEGs. **(C)** Top 5 GO terms and related genes. **(D)** Top 5 GO terms.

Muscle samples from patients with or without cachexia were analyzed for gene expression profile differences. 324 DEGs, including 196 upregulated and 128 downregulated, were illustrated by volcano plot in (**Figure 1A**). The differences between two groups were statistically significant (|logFC| > 0.4, p < 0.05). Between cachectic and non-cachectic groups, the top ten down-regulated DEGs and top ten up-regulated DEGs were listed (**Table 1**). With the purpose of providing a better insight of the potential functional implications of 324 DEGs of muscle between cachectic and non-cachectic groups, GO functional enrichment analysis was performed (**Figure 1B**). Biological process (BP) included neutrophil degranulation, muscle cell migration, platelet-derived growth factor receptor signaling and smooth muscle cell regulation. Cellular components (CC) included membrane region, membrane microdomain, cis-Golgi network and membrane raft. Molecular function (MF) included growth factor binding, pattern binding, polysaccharide binding and transforming growth factor β binding. Specifically, top5 GO terms were displayed (**Figures 1C, D**).

**Table 1:** Top 10 downregulated mRNAs and 10 upregulated mRNAs in cachectic group compared to non-cachectic group.

Gene id	Gene name	log2 Fold Change	*p* value
**Downregulated mRNAs**			
ENSG00000165338	HECTD2-AS1	-0.8858145	0.002022
ENSG00000172456	FGGY-DT	-0.8642546	0.01526
ENSG00000123689	G0S2	-0.8276227	0.000704
ENSG00000118298	CA14	-0.8177314	0.026465
ENSG00000262628	OR1D5	-0.7988045	0.003605
ENSG00000221910	OR2F2	-0.7906266	0.004263
ENSG00000261649	GOLGA6L7	-0.7742982	0.045296
ENSG00000171766	GATM	-0.7541773	0.04579
ENSG00000101542	CDH20	-0.7474618	0.002142
ENSG00000264810	MIR4441	-0.7102938	0.012739
**Upregulated mRNAs**			
ENSG00000166741	NNMT	1.4251374	0.001905
ENSG00000143546	S100A8	1.3080176	0.035033
ENSG00000221716	SNORA11	1.1696882	0.017608
ENSG00000162783	IER5	1.1561934	0.036194
ENSG00000120129	DUSP1	1.0878943	0.021062
ENSG00000123836	PFKFB2	1.0614727	0.002255
ENSG00000200259	SNORD35A	0.9874195	0.001412
ENSG00000162511	LAPTM5	0.9744628	0.002753
ENSG00000134758	RNF138P1	0.9422199	0.01065
ENSG00000173598	NUDT4	0.9301251	0.02745

### GSEA analysis of DEGs in muscle samples with or without cachexia

**Figure 2:**
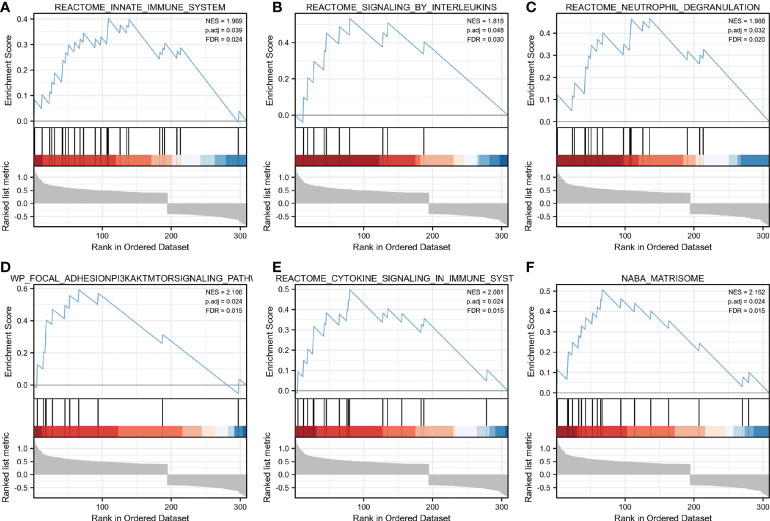
Enrichment plots from GSEA. **(A–F)** Biological pathways involved in skeletal muscle metabolism were illustrated by Gene set enrichment analysis. ES, enrichment score; NES, normalized ES; ADJ P-val, adjusted P-value.

GSEA analysis was performed to identify putative biological pathways involved in skeletal muscle of cachectic and non-cachectic patients. We found that reactome innate immune system, reactome signaling by interleukins, reactome neutrophil degranulation, reactome cytokine signaling in immune system, FAK/PI3K-AKT-mTOR signaling pathway and naba matrisome were enriched in cachectic muscle group as demonstrated in (**Figures 2A-F**). These results demonstrated that immune response and inflammatory cytokines might play critical roles in pathological process of cachexia in skeletal muscle.

### Interaction network of DEGs in muscle samples with or without cachexia

**Figure 3:**
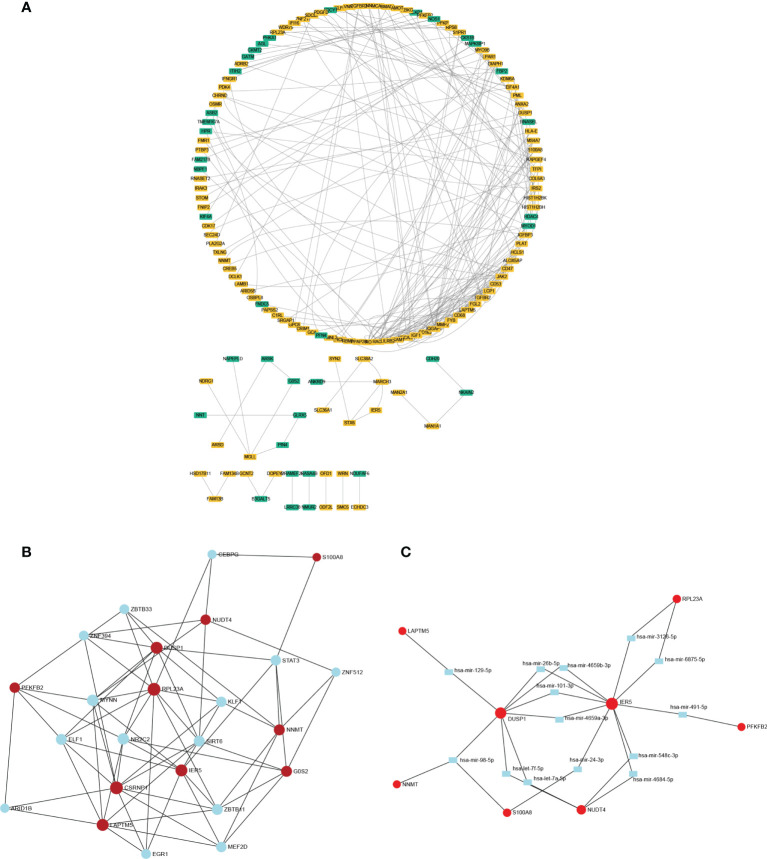
The PPI network and interaction network of DEGs. **(A)** The PPI network of DEGs was constructed through Cytoscape. **(B)** Interaction network linking top 10 DEGs to transcription factor. **(C)** Interaction network linking top 10 DEGs to miRNAs.

The network of DEGs was built in muscle through STRING between cachectic and non-cachectic samples with a threshold of 0.4. It is shown in Cytoscape-MCODE that the PPI network contains 146 nodes and 233 edges (**Figure 3A**). The top 10 DEGs were then linked to potential transcription factors/miRNAs through an interaction network we constructed (**Figures 3B, C**). The genes DUSP1, IER5, and RPL23A were key players in the regulatory network. DUSP1 was associated with transcription factors like ZNF394, ZBTB33, STAT3, SIRT6, MYNN, and KLF1. MiRNAs such as miR-98-5p, miR-4659-3p, miR-101-3p, let-7f-5p, miR-26b-5p, miR-129-5P and let-7a-5p might be regulation factors of DUSP1.

### Muscle atrophy and DUSP1 upregulation induced by cachexia

**Figure 4:**
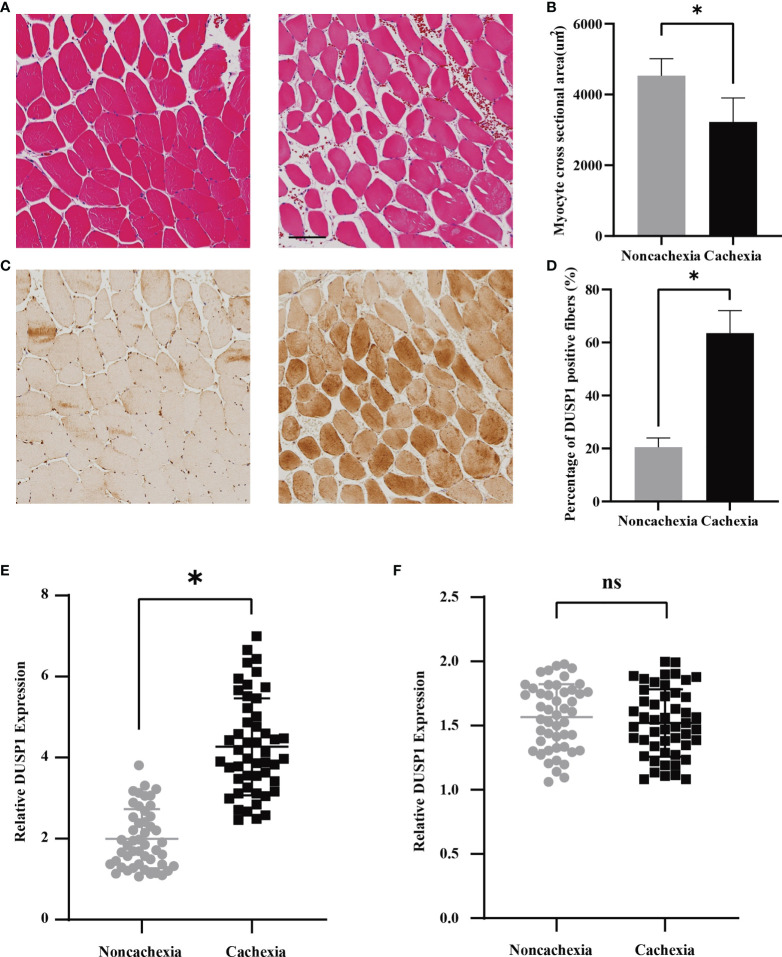
Expression of DUSP1 was associated with pathological process in cancer cachexia. **(A)** Morphological evaluation of skeletal muscle between cachectic and non-cachectic patients (hematoxylin–eosin) (Scale bar=100um). **(B)** Myocyte cross sectional area evaluation between cachectic and non-cachectic patients. **(C)** Representative images of IHC revealing DUSP1 expression between cachectic and non-cachectic patients. **(D)** Percentage of DUSP1 positive fibers in skeletal muscle between cachectic and non-cachectic patients. **(E)** Expression of DUSP1 in muscle tissues between cachectic and non-cachectic patients. **(F)** Expression of DUSP1 in tumor tissues between cachectic and non-cachectic patients. *p < 0.05; ns, not significant.

According to the findings above, we deduced that DUSP1 might act as an essential factor of skeletal muscle atrophy in patients with cancer cachexia. To further clarify clinical significance of DUSP1 in muscle fibers, HE staining, and immunohistochemistry were performed in muscle tissues of rectus abdominis from patients with and without cachexia. Quantitative HE staining showed a significant reduction of myofiber cross-sectional area in the muscles of patients in cachectic and non-cachectic groups in the comparison (**Figures 4A, B**, *p*<0.05). Patients with cachexia had higher levels of DUSP1 expression in their myofibers compared to patients without cachexia, according to representative images of IHC (**Figures 4C, D**, *p*<0.05). Following that, we evaluated the expression of DUSP1 in muscle and tumor tissues of 97 patients with gastrointestinal tumor by qPCR assay. The results showed that cachectic patients exhibited significant DUSP1 upregulation in skeletal muscle tissues (**Figure 4E**), *p*<0.05), while no significant difference of DUSP1 expression in tumor tissues was observed between two groups (**Figure 4F**).

### Correlation of DUSP1 with cachexia-related characteristics

**Figure 5:**
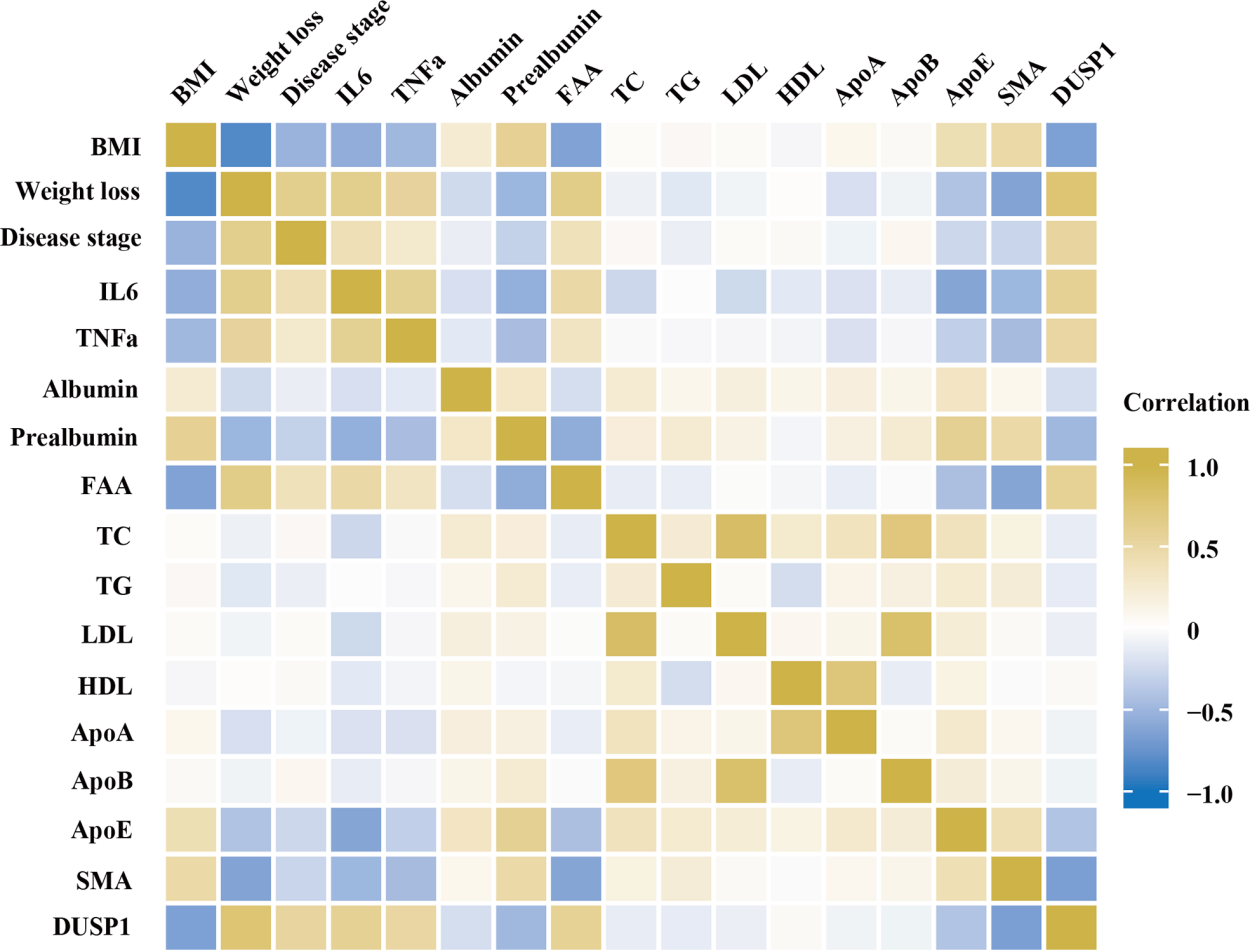
Spearman correlation matrix for variables. Yellow indicates positive correlations and blue indicates inverse correlations. Correlation strength is indicated by color intensity.

The clinical significance of DUSP1 was then investigated in the skeletal muscle tissues of patients with gastrointestinal tumor. All the serum determinations of the 97 patients were evaluated before surgery (**Table 2**). We divided them into cachexia and non-cachexia groups and an analysis of clinical-pathological correlations between groups was conducted. It was showed that cachectic patients had significantly higher level of weight loss, disease stage, IL6, TNFα and free fatty acid (FFA) in the analysis. While they had significantly lower level of BMI, apolipoprotein E, prealbumin and SMA. A correlation matrix illustrated the correlation between cachexia-related clinical-pathological characteristics and DUSP1 expression across all patients. A negative correlation was found between DUSP1 expression and BMI (r=-0.629, *p*<0.01), prealbumin (r=-0.585, *p*<0.01), SMA (r=-0.698, *p*<0.01), and a positive correlation with weight loss (r=0.768, *p*<0.01), IL6 (r=0.750, *p*<0.01) and TNFα (r=-0.575, *p*<0.01), as expected (**Figure 5**). According to these findings, DUSP1 was strongly associated with the muscle atrophy of cachexia in gastrointestinal neoplasms.

**Table 2:** Clinical characteristics of 97 participants.

Clinical characteristics	Cachexia (n = 49)	Non-cachexia (n = 48)	*p* Value
Age	65.10±8.58	62.56±10.51	0.195
BMI	20.54±1.73	24.67±2.47	<0.001^*^
Weight Loss	7.06±2.76	1.01±1.21	<0.001^*^
Disease stage (III/IV)	44/49	13/48	<0.001^*^
IL6 (mmol/L)	9.16±5.52	4.38±1.44	<0.001^*^
TNFa (mmol/L)	13.28±5.17	8.53±2.96	<0.001^*^
Alb (g/L)	39.27±4.12	41.39±4.68	0.190
PAb (mg/L)	196.92±58.43	250.13±40.43	<0.001^*^
FAA(mmol/L)	0.57±0.13	0.35±0.11	<0.001^*^
TC (mmol/L)	4.22±1.01	4.37±0.80	0.390
TG (mmol/L)	1.29±0.38	1.46±0.83	0.063
LDL (mmol/L)	2.55±0.91	2.57±0.71	0.913
HDL (mmol/L)	1.18±0.36	1.16±0.30	0.855
Apo A (g/L)	1.20±0.25	1.28±0.22	0.137
Apo B (g/L)	0.79±0.26	0.78±0.18	0.901
Apo E (mg/L)	37.71±13.25	44.73±12.21	0.008^*^
SMA	116.99±14.08	138.79±13.35	<0.001^*^

BMI, Body mass index; SMA, Smooth muscle area; ALB, Albumin; PAb, Prealbumin; TC, Total cholesterol; TG, Tri-glyceride; LDL, Low-density lipoprotein; HDL, High-density lipoprotein; ApoA, Apolipoprotein A; ApoB, Apolipoprotein B; ApoE, Apolipoprotein E; FFA, Free fatty acid; IL-6, Interleukin 6; TNF-α, Tumor Necrosis Factor-α. *p < 0.05.

### DUSP1 inhibited myoblast differentiation *in vitro*

**Figure 6:**
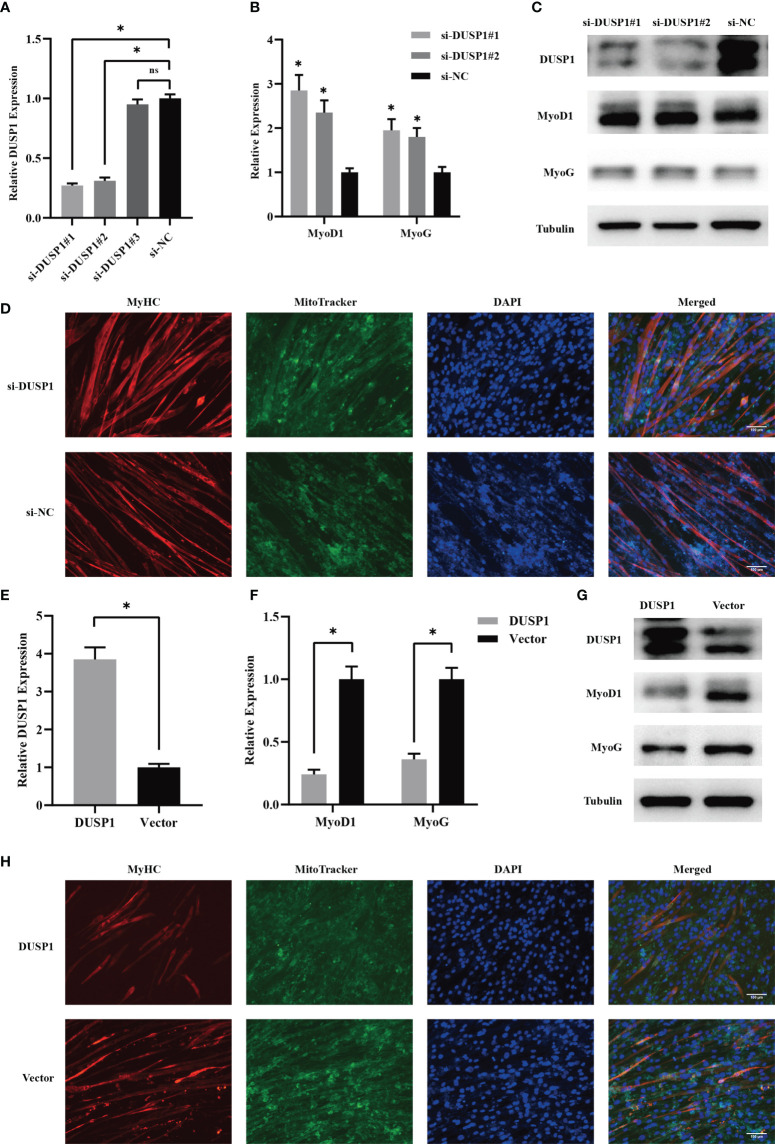
DUSP1 suppressed myoblast differentiation *in vitro*. **(A)** Relative DUSP1 expression with or without DUSP1 knockdown. **(B, C)** Expression of markers in myocyte without or with knockdown of DUSP1 by qPCR and Western blot analysis. **(D)** Representative images of the C2C12 myotube without/with knockdown of DUSP1. Myotube was stained with MyHC and MitoTracker. (Scale bar=100μm). **(E)** Relative DUSP1 expression with or without DUSP1 overexpression. **(F, G)** Expression of markers in myocyte without or with overexpression of DUSP1 by qPCR and Western blot analysis. **(H)** Representative images of the C2C12 myotube without/with overexpression of DUSP1. Myotube was stained with MyHC and MitoTracker. *p < 0.05; ns, not significant.

Due to the importance of myogenesis in maintaining muscle mass, we next investigated how myogenesis is impacted by DUSP1. C2C12 myoblast were transfected with three independent siRNAs before differentiation. Two siRNAs effectively knocked down target genes (**Figure 6A**). The expression of MyoD1 and MyoG increased significantly as a consequence of knockdown of DUSP1 showed in western blotting and qPCR (**Figures 6B, C**). Considering that mitochondrial function might be involved in muscle physiology, immunofluorescence of mitochondria and MyHC in myocyte was performed. The result indicated that mitochondrial morphology revealed fusion-dominant and myogenic differentiation was enhanced after the knockdown of DUSP1 (**Figure 6D**). To further investigate DUSP1’s function in myocytes, we transfected C2C12 with DUSP1 overexpression vectors in order to upregulate expression of DUSP1 before differentiation. The expression of DUSP1 increased about four-fold as a consequence of transfecting of DUSP1 overexpression vector showed in qPCR (**Figure 6E**). When DUSP1 is overexpressed, in both qPCR and western blotting experiments, MyoD1 and MyoG are significantly down-regulated (**Figures 6F, G**). The result of immunofluorescence indicated that mitochondrial morphology revealed fission-dominant and myogenic differentiation was weakened after the overexpression of DUSP1 (**Figure 6H**).

## Discussion

It has been established that muscle atrophy results in a greater risk of adverse outcomes in cachectic patients with gastrointestinal tumor. Due to this, identifying drivers of the muscle atrophy in patients is of utmost importance. In this study, microarray data targeting skeletal muscle from patients with and without cancer cachexia were screened and analyzed. As a result, 128 downregulated and 196 upregulated DEGs differed significantly between cachectic and non-cachectic groups. DUSP1 was one of the most highly expressed genes and act an essential role in regulatory network. In fact, our study found several intriguing correlations between cachexia-related characteristics and DUSP1 expression, providing suggestion that it has functional relevance *in vivo*. Then, *in vitro* experiments showed that DUSP1 could prevent myogenesis by inhibiting myocyte differentiation.

Muscle atrophy attributed to cancer cachexia is perceived as the result of dysfunction in multiple signaling pathways regulating protein synthesis and proteolysis (17). Specifically, it has been demonstrated that factors derived from tumors induce skeletal muscle atrophy by activating NF-kB and proteolysis of specific muscle mediated by ubiquitin proteasome pathway (UPS) in studies with mouse Lewis lung carcinoma (LLC) and C26-colon carcinoma (C26) (18). Besides proteolysis, impaired myogenesis and regulatory dysfunction are implicated in cachexia-induced loss of skeletal muscle (19). Since protein turnover is so low that a small imbalance can lead to significant muscle loss, cachexia-induced muscle atrophy is hard to treat and results in a much higher mortality (20–22). Therefore, we aimed to identify some DEGs that had played crucial roles in the muscle atrophy in patients with cachexia. Microarray data including 5 non-cachectic patients and 17 cachectic patients from GEO database were analyzed. Among 324 DEGs, 196 of them were upregulated and 128 of them were downregulated in rectus abdominis biopsies of patients with cachexia. With the result of GSEA analysis and DEGs interaction network, we concluded that DUSP1 might be crucial in the pathological process of muscle atrophy in cachectic patients.

It was well established that the ubiquitin proteasome pathway critically contributes to the initiation and development of cancer cachexia. As consequence of activation, increased expression of muscle atrophy F-box (MAFbx/FbxO32) in muscles leads to a higher skeletal muscle degradation. In addition, MyHC, MyoD, and MyoG, related to myogenesis, were downregulated and myostatin (Mstn) was upregulated (23, 24).

DUSP1 belongs to superfamily of protein tyrosine phosphatases that includes 10 MAPK phosphatases (MKPs) that inhibit MAPK signaling through dephosphorylation of MAPKs (25). It has been established that DUSP1,among the 10 family members, plays a regulatory role in a variety of cellular responses, especially in inflammation. Prior reports have indicated that DUSP1 regulates oral cancer-associated inflammation and leukocyte infiltration (26). However, to date, the role of DUSP1 in muscle atrophy of cancer cachexia remains unclear. As far as we know, it has been documented that DUSP1 could influence the differentiation of skeletal myocyte *via* negatively modulating the ERK/MAPK pathway (27). But the expression and function of DUSP1 in muscle atrophy during cancer cachexia has not been reported. A significant upregulation of DUSP1 was found in skeletal muscle of cachectic patients in our study. DUSP1 expression in cachectic group was found to have negative correlation with SMA, prealbumin and BMI and positive correlation with TNFα, IL6 and weight loss. These findings indicated that DUSP1 gene contributes significantly to the muscle atrophy during cancer cachexia. Our study then confirmed that upregulation of DUSP1 inhibited MyoD1, MyoG and MyHC expression in myocyte, along with the mitochondrial fission. Inhibition of myocyte differentiation induced by malfunction of mitochondria was reported to be an effector of muscle atrophy in cancer cachexia (28). Previously, our study has observed some abnormal mitochondrial fission in skeleton muscle cells of cachectic patients, which provided additional insights into the dynamics of mitochondria in cachectic muscle atrophy (8). By overexpressing and knocking down DUSP1, our study clarified that DUSP1 induces muscle atrophy by inhibiting myogenesis in skeletal muscle. Consistent with our previous study, these indicated that myogenesis-related genes might be important regulators of the muscle synthesis in cancer cachexia. Previous research has proved that angiotensin II could lead to weight loss through increased protein breakdown, reduced protein synthesis in skeletal muscle through ERK/MAPK pathway (29). It is deserved to explore whether angiotensin II could be regulated by DUSP1 through ERK/MAPK pathway in further research.

There are limitations to this study. Although expression of DUSP1 did not reveal significant deference in tumor tissues between patients with and without cachexia, we cannot confirm whether DUSP1 in skeletal muscle was regulated by tumor-derived factors. Further research will be needed to investigate the connections between tumor-derived factors and DUSP1.

## Conclusions

In conclusion, our study screened gene expression profiles of skeletal muscle in patients with and without cancer cachexia. Numerous DEGs have been identified and DUSP1 was one of the highly expressed genes. Clinical studies showed that DUSP1 expression was found to have negative correlation with SMA, prealbumin and BMI and positive correlation with TNFα, IL6 and weight loss. *In vitro* experiments demonstrated that DUSP1 hindered myogenesis through modulation of differentiation related genes. As a result of our findings, DUSP1 was expected to be a specific target for preventing and treating cachectic muscle atrophy in patients with gastrointestinal tumor.

## Data availability statement

The datasets presented in this study can be found in online repositories. The names of the repository/repositories and accession number(s) can be found in the article/**Supplementary Material**.

## Ethics statement

The studies involving human participants were reviewed and approved by ethics committee of Zhongshan Hospital, Fudan University. The patients/participants provided their written informed consent to participate in this study. The animal study was reviewed and approved by The Animal Care Committee of Fudan University.

## Author contributions

XS and GW conceived, designed, and drafted the manuscript. QM and XM collected the clinical data. XS, QM, and XM performed the experiments. XS and QM conducted the statistical analyses. XS drafted the manuscript. GW and XS revised the manuscript. All authors contributed to the article and approved the submitted version.

## Funding

This study was supported by Shanghai Health Commission-Clinical Nutrition (2019ZB0105) and Clinical Research Special Fund of Zhongshan Hospital, Fudan University (2020ZSLC17).

## Acknowledgments

We are grateful to Prof. Ying Feng from Shanghai Institute of Nutrition and Health, Chinese Academy of Sciences for kindly providing the C2C12 myoblast.

## Conflict of interest

The authors declare that the research was conducted in the absence of any commercial or financial relationships that could be construed as a potential conflict of interest.

## Publisher’s note

All claims expressed in this article are solely those of the authors and do not necessarily represent those of their affiliated organizations, or those of the publisher, the editors and the reviewers. Any product that may be evaluated in this article, or claim that may be made by its manufacturer, is not guaranteed or endorsed by the publisher.

## Abbreviation

DEG, differentially expressed gene; DUSP1, dual specificity phosphatase; SMA, skeletal muscle area; TNF, tumor necrosis factor; IL-6, Interleukin-6; MAPK, mitogen-activated protein kinase; GEO, gene expression omnibus; PPI, protein-protein interaction; GSEA, gene set enrichment analysis; MyoD1, myogenic differentiation 1; MyoG, myogenin; CSA, cross sectional area; MyHC, myosin heavy chain; BP, biological process; CC, cellular components; MF, molecular function; FFA, free fatty acid; UPS, ubiquitin proteasome pathway; LLC, Lewis lung carcinoma; ES, enrichment score.
