# Supplementary material for: DUSP1 promotes muscle atrophy by inhibiting myocyte differentiation in cachectic patients
Source: Front Oncol. 2022 Nov 1;12:1040112. doi: 10.3389/fonc.2022.1040112 (PMC9663480; doi:10.3389/fonc.2022.1040112)
Supplement: Supplementary file 1 [file Table_1.docx]

**Table S1. Primer Sequences used for qRT-PCR.**

| Name |  | Primer Sequences |
| --- | --- | --- |
| DUSP1 | Forward | AGGCCATTGACTTCATAGACTC |
|  | Reverse | AGTCCTCATAAGGTAAGCAAGG |
| Dusp1 | Forward | TAACCACTTTGAGGGTCACTAC |
|  | Reverse | GAAGTCAATAGCCTCGTTGAAC |
| MyoD | Forward | ACTTCTATGATGACCCGTGTTT |
|  | Reverse | ACATGCTCATCCTCACGAG |
| Myogenin | Forward | AACCCAGGAGATCATTTGCTC |
|  | Reverse | GAAGGCAACAGACATATCCTCC |
| GAPDH | Forward | TTGCCCTCAACGACCACTTT |
|  | Reverse | TGGTCCAGGGGTCTTACTCC |
| si-Dusp1#1 | Forward | GUGCCCUGAACUACCUUAATT |
|  | Reverse: | UUAAGGUAGUUCAGGGCACTT |
| si-Dusp1#2 | Forward: | GCUCCUUCUUCGCUUUCAATT |
|  | Reverse: | UUGAAAGCGAAGAAGGAGCTT |
| si-Dusp1#3 | Forward: | CCACUCAAGUCUUCUUUCUTT |
|  | Reverse: | AGAAAGAAGACUUGAGUGGTT |
